# β_2_-Adrenergic Receptor Activation Suppresses the Rat Phenethylamine Hallucinogen-Induced Head Twitch Response: Hallucinogen-Induced Excitatory Post-synaptic Potentials as a Potential Substrate

**DOI:** 10.3389/fphar.2018.00089

**Published:** 2018-02-08

**Authors:** Gerard J. Marek, Brian P. Ramos

**Affiliations:** ^1^Department of Psychiatry, School of Medicine, Ribicoff Research Facilities of the Connecticut Mental Health Center, Yale University, New Haven, CT, United States; ^2^Astellas Pharma Global Development, Inc., Global Medical Science, CNS and Pain, Northbrook, IL, United States; ^3^Department of Neurobiology, School of Medicine, Yale University, New Haven, CT, United States

**Keywords:** 2,5-dimethoxy-4-iodoamphetamine (DOI), head twitch response (HTR), epinephrine, layer V pyramidal neurons, medial prefrontal cortex (mPFC), excitatory post-synaptic potentials (EPSPs), clenbuterol, ICI-118,551

## Abstract

5-Hydroxytryptamine_2A_ (5-HT_2A_) receptors are enriched in layers I and Va of the rat prefrontal cortex and neocortex and their activation increases the frequency of glutamatergic excitatory post-synaptic potentials/currents (EPSP/Cs) onto layer V pyramidal cells. A number of other G-protein coupled receptors (GPCRs) are also enriched in cortical layers I and Va and either induce (α_1_-adrenergic and orexin_2_) or suppress (metabotropic glutamate_2_ [mGlu_2_], adenosine A_1_, μ-opioid) both 5-HT-induced EPSCs and head twitches or head shakes induced by the phenethylamine hallucinogen 2,5-dimethoxy-4-iodoamphetamine (DOI). Another neurotransmitter receptor also localized to apparent thalamocortical afferents to layers I and Va of the rat prefrontal cortex and neocortex is the β_2_-adrenergic receptor. Therefore, we conducted preliminary electrophysiological experiments with rat brain slices examining the effects of epinephrine on electrically-evoked EPSPs following bath application of DOI (3 μM). Epinephrine (0.3–10 μM) suppressed the late EPSPs produced by electrical stimulation and DOI. The selective β_2_-adrenergic receptor antagonist ICI-118,551 (300 nM) resulted in a rightward shift of the epinephrine concentration-response relationship. We also tested the selective β_2_-adrenergic receptor agonist clenbuterol and the antagonist ICI-118,551 on DOI-induced head twitches. Clenbuterol (0.3–3 mg/kg, i.p.) suppressed DOI (1.25 mg/kg, i.p.)-induced head twitches. This clenbuterol effect appeared to be at least partially reversed by the selective β_2_-adrenergic receptor antagonist ICI-118,553 (0.01–1 mg/kg, i.p.), with significant reversal at doses of 0.1 and 1 mg/kg. Thus, β_2_-adrenergic receptor activation reverses the effects of phenethylamine hallucinogens in the rat prefrontal cortex. While G_i_/G_o_-coupled GPCRs have previously been shown to suppress both the electrophysiological and behavioral effects of 5-HT_2A_ receptor activation in the mPFC, the present work appears to extend this suppressant action to a G_s_-coupled GPCR. Furthermore, the modulation of 5-HT_2A_ receptor activation-induced glutamate release onto mPFC layer V pyramidal neurons apical dendrites by a range GPCRs in rat brain slices appears to results in behaviorally salient effects of relevance when screening for novel CNS therapeutic drugs.

## Introduction

The common pharmacological action shared between the three major classes of serotonergic hallucinogens (indoleamines [e.g., psilocybin], phenethylamines [e.g., mescaline or DOI], and ergots [e.g., lysergic acid diethylamide (LSD)] is activation of the 5-HT_2_ family of receptors (Nichols, 2016). Activation of 5-HT_2A_ receptors appears to mediate these psychotomimetic effects in humans and psychotomimetic-like effects in rodents (Canal and Morgan, 2012; Kometer and Vollenweider, 2016). The head twitch response induced by serotonergic hallucinogens in rodents frequently has been used as a psychotomimetic-like screen for potential antipsychotic drugs as part of a broader effort to understand psychotomimetic effects of serotonergic hallucinogens, NMDA receptor antagonists, and amphetamine. Conversely, blockade of 5-HT_2A_ receptors may be involved in both the antipsychotic and antidepressant effects of many psychotherapeutic drugs, which implies that screening compounds on DOI-induced head twitches in rodents may have a broader significance than simply the treatment of psychosis (Marek et al., 1992, 2003). The role played by 5-HT_2A_ receptors in modulating thalamocortical circuitry intimately related to arousal and attention may explain the diverse nature of effects seen by both activation and blockade of 5-HT_2A_ receptors (Berendse and Groenewegen, 1991; Marek et al., 2001; Muller et al., 2017).

Regarding the circuitry of serotonergic hallucinogens in the prefrontal cortex (PFC)/neocortex, one prominent effect of 5-HT_2A_ receptor activation in the PFC/neocortex is to increase the frequency of spontaneous 5-HT-induced EPSCs when recording from layer V pyramidal cells with slice preparations from adult rats (Aghajanian and Marek, 1997). While most 5-HT_2A_ receptors in the PFC would appear to be post-synaptic in pyramidal cells, activation of a minority of medial prefrontal coretex (mPFC) 5-HT_2A_ receptors appears to induce these spontaneous EPSCs from thalamocortical afferents, likely arising from the midline and intralaminar thalamic nuclei (Lambe and Aghajanian, 2001; Marek et al., 2001). A number of generally predominantly presynaptic receptors (μ-opiate, mGlu_2_, mGlu_4_, adenosine A_1_) suppress these 5-HT-induced EPSCs (Marek and Aghajanian, 1998a; Marek et al., 2000; Stutzman et al., 2001; Zhang and Marek, 2007). These effects appear to be analogous to the presynaptic effect of orexin_2_ receptor agonists to increase the frequency of spontaneous EPSCs from thalamocortical afferents arising from the midline and intralaminar thalamic nuclei (Lambe and Aghajanian, 2003). Activation of 5-HT_2A_ receptors in PFC slice preparations enhances synaptic overflow of glutamate (Aghajanian and Marek, 1999; Lambe and Aghajanian, 2006; Aghajanian, 2009). Stimulation of the white matter deep to the cortex or in layer V coincident with application of DOI to the slice induces late EPSPs with different pharmacological sensitivity than both the evoked early EPSPs and polysynaptic EPSPs. 5-HT, itself, completely suppresses the evoked late EPSP elicited by stimulation of the white matter deep to the cortex combined with bath application of the phenethylamine hallucinogen DOI (Aghajanian and Marek, 1999). In contrast, the evoked early EPSP elicited under these conditions is minimally suppressed by several agonists which completely suppresses the evoked late EPSP. G*_i_*/G*_o_*-coupled GPCR receptor activation (e.g., mGlu2, adenosine A_1_) also suppresses the evoked late EPSPs with greater potency and efficacy than the evoked early EPSCs. Thus, there appears to be some similar pharmacological sensitivity of the evoked late EPSPs compared to the spontaneous EPSCs induced by 5-HT_2A_ receptor activation, though there are also differences as activation of NMDA NR_2*B*_ receptors appears to play a permissive role in mediating the evoked late EPSCs (Lambe and Aghajanian, 2006).

Dopamine was previously found to suppress glutamate overflow in the PFC evoked by the combination of electrical stimulation and DOI, via dopamine D_1_/D_5_ receptors and the canonical cAMP transduction pathway (Lambe and Aghajanian, 2007). These effects are consistent with preliminary experiments testing epinephrine for efficacy in suppressing late EPSCs evoked by electrical stimulation and DOI while recording from layer V pyramidal cells of the mPFC. Agonists for μ-opiate, mGlu_2_, mGlu_4_, and adenosine A_1_ receptors suppress both electrophysiological effects and a behavior induced by 5-HT_2A_ receptor activation (DOI-induced head twitches) that are mediated in part by output from the mPFC (Gewirtz and Marek, 2000; Klodzinska et al., 2002; Marek, 2003, 2009; Benneyworth et al., 2007; Wieronska et al., 2012; Slawinska et al., 2013). With this background, these preliminary electrophysiological experiments led to testing both the β_2_-adrenergic receptor agonist clenbuterol (Ordway et al., 1987) and the antagonist ICI-118,551 (Beer et al., 1988) in modulating DOI-induced head shakes in rats. The results of these experiments are consistent with the hypothesis that activation of β_2_-adrenergic receptors suppresses glutamate release induced by 5-HT_2A_ receptor activation in the PFC.

## Materials and Methods

### Electrophysiology

Brain slices were prepared from male Sprague–Dawley rats (Harlan, Indianapolis, IN, United States; 120–200 g; *n* = 10) as described previously (Aghajanian and Rasmussen, 1989). Briefly, rats were anesthetized with chloral hydrate (400 mg/kg, i.p.) and decapitated. Coronal slices (500 μm) were cut with an oscillating-blade tissue slicer at a level corresponding to approximately 2.5 mm anterior to bregma. A slice containing the mPFC was then transferred to the stage of a fluid-gas interface chamber which had a constant flow of humidified 95% O_2_, 5% CO_2_. The slices were perfused in a chamber heated to 34°C with normal ACSF which consisted of (in mM) NaCl 126; KCl 3; CaCl_2_ 2; MgSO_4_ 2; NaHCO_3_ 26; NaH_2_PO_4_ 1.25; and D-glucose 10.

Intracellular recording and single-electrode voltage clamping were conducted in layer V pyramidal cells using an Axoclamp-2A (Axon Instruments, Inc., Foster City, CA, United States) as previously described (Aghajanian and Marek, 1997). Stubby electrodes (∼8 mm, shank to tip) with relatively low capacitance and resistance (30–60 MΩ) were filled with 1 M potassium acetate. Layer V pyramidal cells were recorded in current clamp mode. Evoked potentials were obtained while holding cells at -80 mV and stimulating the forceps minor in the white matter deep to the cortex with a bipolar tungsten electrode.

To uncover optimal stimulating sites, 5-HT (100 μM) was bath-applied while stimulating the forceps minor at a 0.1 Hz. 5-HT was turned off within 1–2 min while stimulation continued. Several minutes after turning off the 5-HT bath application, late EPSPs emerged in a fraction of cells. In those pyramidal cells in which late EPSPs did not emerge, the stimulating site was changed. In those cells in which late EPSPs emerged during the 5-HT washout period in response to the low-frequency stimulation, the combination of 0.1 Hz stimulation and DOI also evoked late EPSPs. In those cells in which late EPSPs did not emerge during the 5-HT washout period in response to the low-frequency stimulation, the combination of 0.1 Hz stimulation and DOI also did not evoke late EPSPs.

For those cells with late EPSPs induced by the combination of DOI and white matter stimulation, either a single concentration of epinephrine or an epinephrine concentration–response relationship was tested. The effect of epinephrine on the frequency of late EPSPs during 10 consecutive electrical stimulations (0.1 Hz) as well as the maximal amplitude (mV) of early and late EPSPs was measured. For these experiments, each concentration of epinephrine was bath-applied for 1 min prior and during stimulation of the deep white matter 10 times every 10 s. A 6–8 min washout interval was used between successively higher epinephrine concentrations. For a subset of these cells, blockade of the suppressant effect by epinephrine was attempted with the selective β_2_-adrenergic receptor antagonist ICI-118,551 (300 nM applied initially for 10 min and between successive epinephrine administrations). EC_50_s for the epinephrine concentration-response curves were calculated with GraphPad Prism. The pA_2_ value for ICI-118,551 was calculated by using the formula derived by Arunlakshana and Schild (1959); *p*A_2_ = log [Antagonist] – log (dr -1) where dose ratio (dr) was the EC_50_ for epinephrine in the presence of the ICI-118,551 divided by the EC_50_ for norepinephrine in the absence of ICI-118,551. The apparent K*_b_* value for ICI-118,551 was calculated from the pA_2_ value (Arunlakshana and Schild, 1959).

### Behavioral Experiments

Male Sprague–Dawley rats (*n* = 112) weighing between 150 and 300 g at the initial behavioral testing were used (Harlan, Indianapolis, IN, United States). They were housed in suspended stainless wire cages (18 cm × 36 cm × 20 cm) with two to four rats occupying each cage. The colony room was maintained at 20°C and relative humidity (60%). The room was illuminated 12 h/day (07:00–19:00). All rats had free access to laboratory chow (Teklad 4% Rat Diet) and water except during experimental sessions. All animals were treated in accord with the National Institutes of Health’s Guide for the Care and Use of Laboratory Animals. In addition, all protocols were approved by the Yale University Animal Care and Use Committee. All experiments were performed between 09:00 and 16:00. The animals were transferred to a clear polycarbonate cage (43 cm × 21.5 cm × 20 cm) with a sawdust-covered floor. All the rats were habituated to the testing environment with a saline injection at least several days prior to the first DOI/vehicle, DOI and clenbuterol/vehicle, or DOI/clenbuterol/ICI-188,551/vehicle injection. Clenbuterol (0.3–3 mg/kg, i.p.) was administered 15 min prior to DOI. The β_2_-adrenergic antagonist ICI-118,551 (0.01–1 mg/kg, i.p. was injected 30 min prior to DOI. The animals were observed during consecutive 5 min periods for a total of 30 min following the DOI injection. The observations began 1 min after injecting DOI to allow time for drug absorption. In addition to counting each head shake response, forward locomotion (movement from one end to the other end of the cage was scored as one cross) and rearing (rising up on hind limbs) were recorded.

### Statistical Analysis

A two-way ANOVA was carried out for the electrophysiological data to compare the epinephrine concentration–response curve on the early EPSPs vs. the late EPSPs. The late EPSPs data (*n* = 6) was analyzed with a one-factor ANOVA and comparisons to the control condition were made with the Dunnett test (*p* < 0.05).

A one-factor ANOVA was carried out for measurement of head shakes, rearing, and horizontal locomotor activity. The Dunnett test was used to test for specific comparisons to the DOI alone or the DOI/clenbuterol group, respectively. The effect of the β_2_-adrenergic receptor agonist clenbuterol was assessed using a between-subject design where a different cohort of rats was used for each dose (*n* = 12/group). The effect of the β_2_-adrenergic receptor antagonist ICI-118,559/vehicle with DOI/vehicle also was tested using a between-subject design (*n* = 16/group) in a separate second behavioral experiment. A slightly higher sample size was used for the β_2_-adrenergic antagonist experiment given the uncertainty regarding the occupancy of β_2_-adrenergic receptor at the doses chosen. The level of significance was set for *p* < 0.05.

### Drugs

Doses were calculated based on the salt forms. The drugs were dissolved in saline, neutralized to a pH of ∼7.4, and injected i.p. in a volume of 1 ml/kg body weight. The β_2_-adrenergic receptor agonist clenbuterol and the β_2_-adrenergic receptor antagonist ICI-118,559 were purchased from Tocris (Ballwin, MO) and Research Biochemical International (RBI; Natick, MA, United States), respectively. The 5-HT_2A/2B/2C_ receptor partial agonist DOI, (±)-2,5-dimethoxy-4-iodoamphetamine hydrochloride, was purchased from RBI. A dose of DOI (1.25 mg/kg, i.p.) producing a near-maximum of head shakes over a 30 min period was chosen for experiments testing suppression of head shakes by clenbuterol (Gewirtz and Marek, 2000). DOI-induced head shakes increase in a monotonic dose-dependent manner through 9 mg/kg using Sprague–Dawley rats (Pranzatelli, 1990). For the electrophysiological experiments, (-) epinephrine bitartrate was purchased from Sigma (St. Louis, MO, United States). A 1 M stock solution was prepared with 10 mM sodium metabisulfite and diluted appropriately for the final solution bath applied to the slice. 5-Hydroxytryptamine (5-HT) creatine sulfate was purchased from Sigma Chemical, Co. (St. Louis, MO, United States). 5-HT was used only to find cells in which 5-HT_2A_ receptor stimulation would induce late EPSCs.

## Results

Layer V pyramidal cells of the mPFC (predominantly in the prelimbic area; Cg3) were recorded in a zone *ca* 1/2–2/3 the distance between the pial surface and the subcortical white matter. The pyramidal cells in the present study had the following characteristics similar to that reported previously using this methodology with a resting potential of ∼71 mV; an action potential amplitude of ∼82 mV; an action potential duration of ∼0.8 ms (at half-amplitude); and an input resistance for a 0.4 nA test-pulse of ∼35 MΩ.

### Epinephrine Selectively Suppresses Electrically-Evoked Late EPSPS after DOI

**FIGURE 1:**
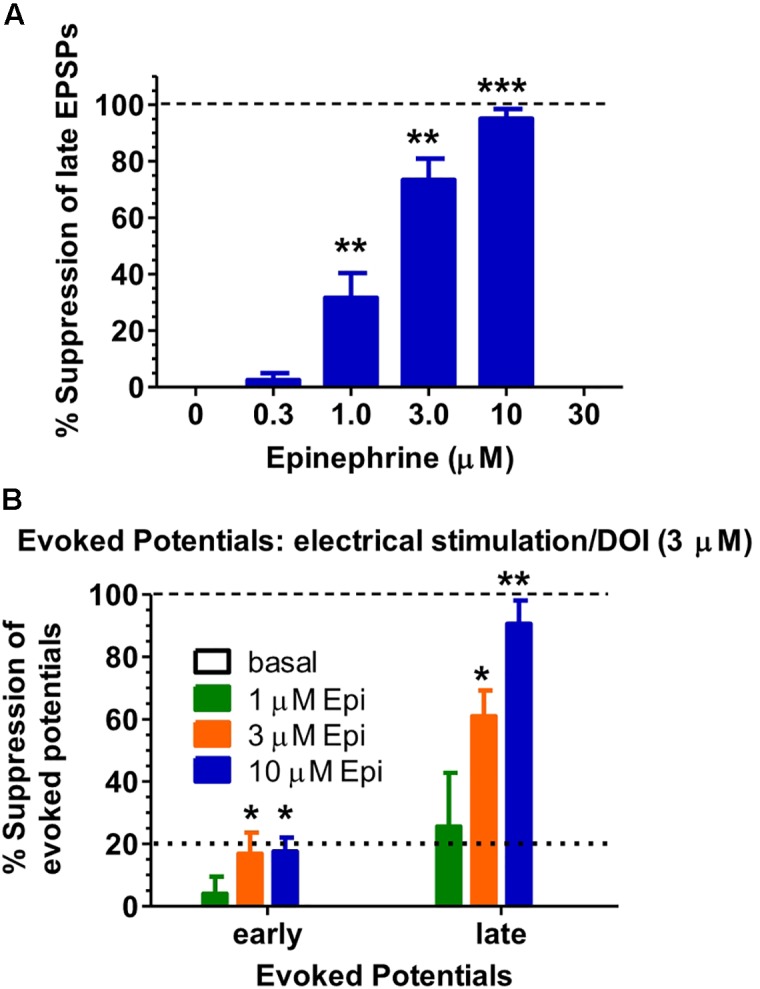
Epinephrine selectively suppresses late EPSPs evoked by electrical stimulation/DOI. **(A**, top graph) Epinephrine (0.3–10 μM), in a monotonic dose-dependent manner, suppresses the frequency of late EPSPs induced by electrical stimulation (0.1 Hz × 10 Hz) after application of DOI (3 μM × 10 min) to rat medial prefrontal cortex (mPFC) slices (*n* = 6). **(B**, bottom graph) Epinephrine (1–10 μM) selectively suppresses the amplitude of late EPSPs evoked by electrical stimulation (0.1 Hz × 10) after application of DOI (3 μM × 10 min) in slices from three of the six rats shown above. While epinephrine did significantly suppress the early EPSPs at the two higher concentrations, epinephrine did not suppress the early EPSP induced by electrical stimulation after DOI by more than 29% in any of the 3 cells at any epinephrine concentration. ^∗^*p* < 0.05, ^∗∗^*p* < 0.01, ^∗∗∗^*p* < 0.001 compared to control using the Dunnett test.

**FIGURE 2:**
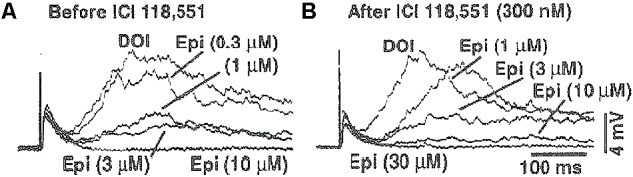
Epinephrine selectively suppressed late EPSPs evoked by electrical stimulation/DOI with a rightward shift in the epinephrine concentration-response curve by the β_2_-adrenergic receptor antagonist ICI-118.551. **(A)** Epinephrine (0.3–10 μM) bath application suppressed the amplitude of DOI/electrically-evoked late EPSPs in a concentration-dependent manner in a cell from the rat mPFC. **(B)** In the same cell shown in **(A)**, the β_2_-adrenergic receptor antagonist ICI-118,551 (300 nM × 10 min pretreatment, during and between epinephrine bath applications) induced a rightward shift of the epinephrine concentration-response curve with a pA_2_ of 7.37 (apparent K_b_ of 43 nM).

Epinephrine (10 μM, applied for 1 min before 10 successive stimulations of the white matter every 10 s) suppressed late EPSCs following DOI (3 μM × 10–15 min) either nearly completely or completely in 10 consecutive cells from 10 different rats. Epinephrine (0.3–10 μM) suppressed, in a monotonic concentration-dependent manner, the frequency of electrically-evoked late EPSPs following bath application of DOI (3 μM) to the slice for 10 min (n = 6, **Figures 1A**, **2**). The EC_50_ for epinephrine in suppressing the frequency of late EPSPs (in an all-or-none fashion) was 1.65 μM. The one-factor ANOVA for the frequencies of late EPSCs was significant [F(3,6) = 11.35, p = 0.0069]. The 1, 3, and 10 μM epinephrine concentrations showed significant suppression of the late EPSCs compared to the control condition (p < 0.05).

Measurement of both the electrically-evoked early EPSP and the late EPSP amplitude after DOI demonstrated the selectivity of epinephrine for suppression of the late EPSPs in a subset of these cells (*n* = 3; **Figure 1B**) as epinephrine suppressed the early EPSP by 18% compared to a maximal ∼91% suppression of the late EPSP at 10 μM. The EC_50_ for epinephrine in suppressing the maximal amplitude of late EPSPs was 2.06 μM. The mean basal magnitude of the electrically-evoked early EPSP and late EPSP after DOI administration was 5.3 (± 1.2, SD) and 9.8 (± 2.3, SD) mV. While significant effects were observed for time factor of early vs. late EPSCs [*F*(1,8) = 38.42, *p* = 0.0003] and the epinephrine concentration factor [*F*(3,8) = 17.75, *p* = 0.0007], the robust effect of epinephrine on late EPSCs is reflected in the significant interaction between the time and epinephrine concentration factors [*F*(3,8) = 7.80, *p* = 0.0092].

**FIGURE 3:**
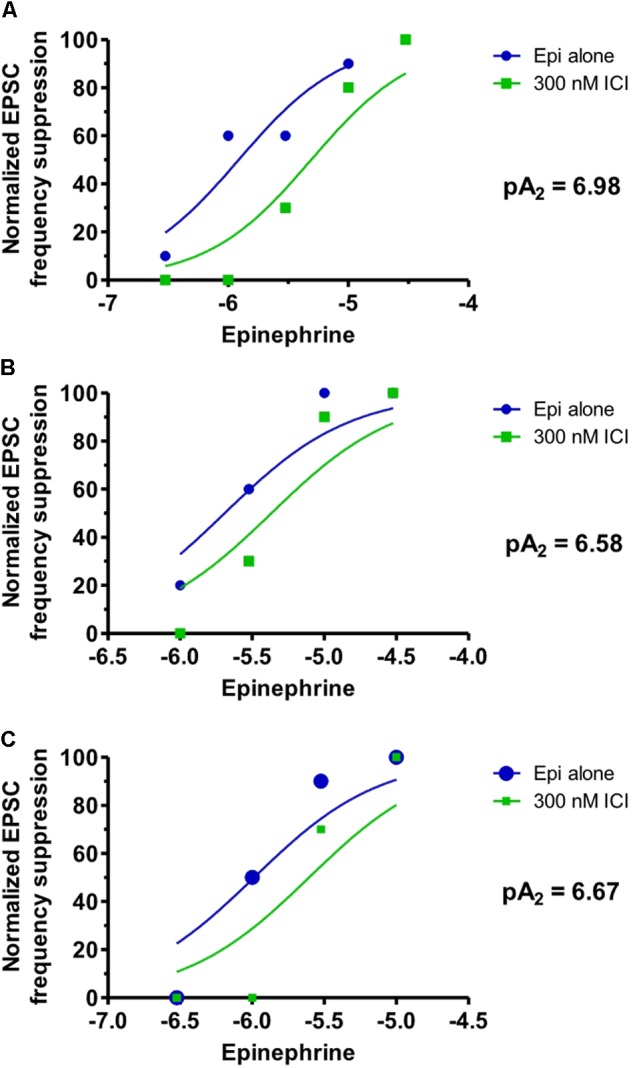
ICI-118,551 blocked the suppressant action of epinephrine on late EPSPs evoked by electrical stimulation/DOI. The shift in the epinephrine concentration-response suppression in the frequency of late EPSPs induced by electrical stimulation/DOI is shown for three cells **(A–C)** before and during bath application of the β-adrenergic receptor antagonist ICI-118,551 (300 nM). The pA_2_ calculated for each cell using the Schild equation is displayed for each cell.

Experiments were conducted in three cells where the frequency of the late EPSCs were measured to suggest whether or not β_2_-adrenergic receptor activation might be mediating these effects of epinephrine. Bath application of ICI-118,551 (300 nM) blocked the suppressant action of epinephrine on the electrically-evoked late EPSP after DOI in three cells (**Figure 3**) with a mean pA_2_ value of 6.74 (SEM of 0.12). The apparent K*_b_* in these three cells ranged from 105 to 217 nM. In two of these cells, the maximal amplitude of the late EPSCs was also measured. The pA_2_ calculated from the Schild equation was 7.37 and 6.22 in these two cells where the pA_2_ determined from the late EPSC frequency analysis was 6.98 and 6.58 respectively. The cell with a pA_2_ of 7.37 and apparent K_b_ of 43 nM from the late EPSP amplitude analysis is shown in **Figure 2**.

### DOI-Induced Head Twitches

**FIGURE 4:**
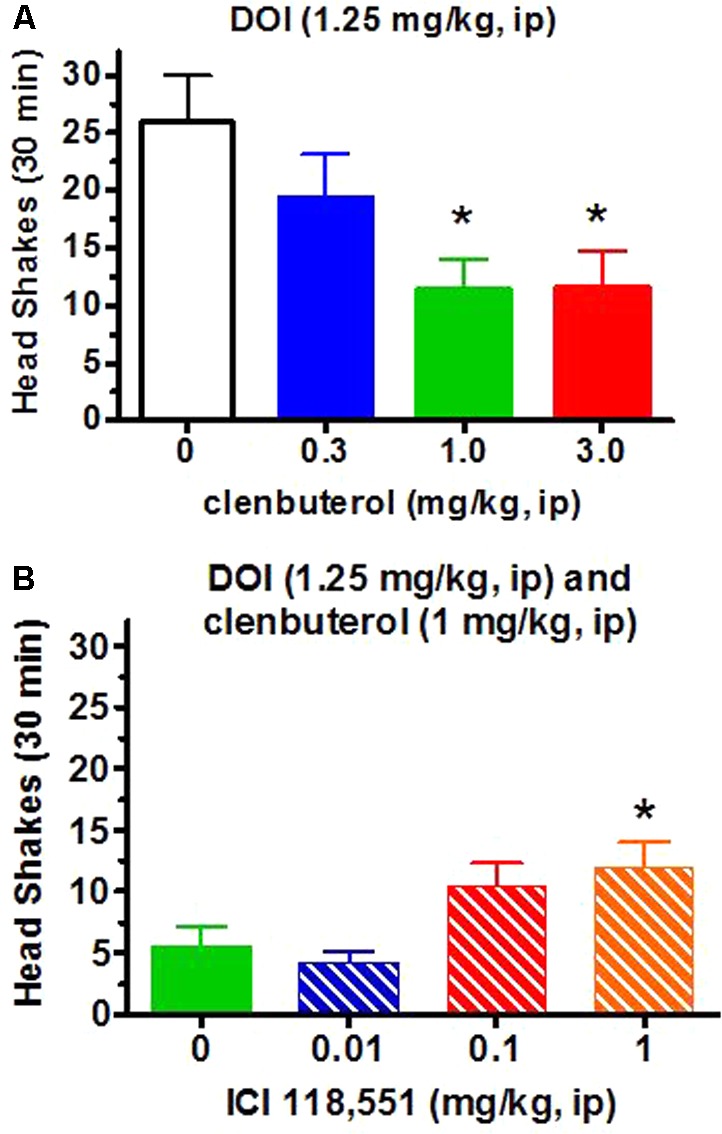
Clenbuterol (0.3–3.0 mg/kg, i.p.), via β_2_-adrenergic receptor activation, suppresses DOI (1.25 mg/kg, i.p.)-induced head shakes. (**A**, top graph) A dose-dependent suppression of DOI-induced head shakes is observed with significant suppression by clenbuterol of approximately 55% at the 1.0 and 3.0 mg/kg doses (*n* = 12/group). (**B**, bottom graph) In an entirely independent experiment, the β_2_-adrenergic receptor antagonist ICI-118,551 blocked the clenbuterol (1.0 mg/kg)-induced suppression of DOI (1.25 mg/kg)-induced head twitches in a dose-dependent manner with a significant effect at 1.0 mg/kg ICI-118,551 (*n* = 16/group). ^∗^*p* < 0.05 for comparison to DOI/clenbuterol vehicle or in comparison to DOI/clenbuterol/ICI-118,551 vehicle (bottom graph).

**FIGURE 5:**
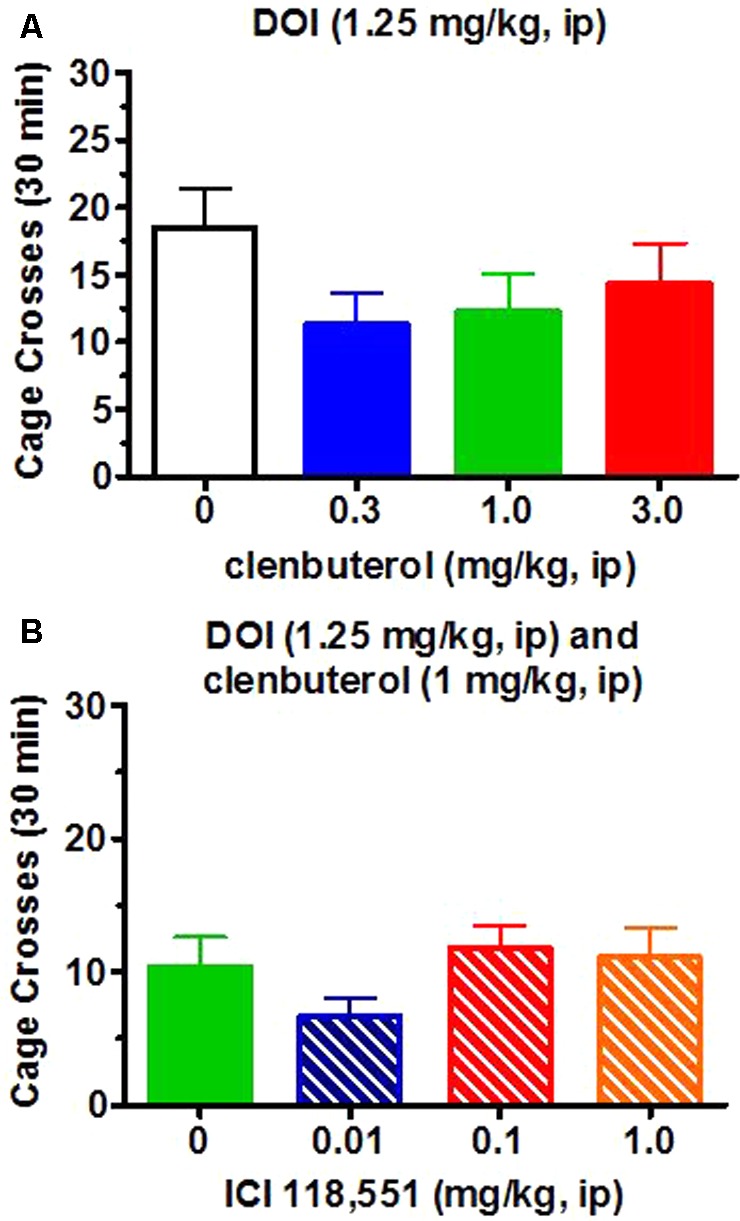
Neither clenbuterol (0.3–3.0 mg/kg, i.p.) or ICI-118,551 (0.1–1.0 mg/kg) altered horizontal locomotor activity after DOI. (**A**, top graph) No significant changes were observed in the frequency cage crosses over a 30 min period following vehicle or clenbuterol and DOI. (**B**, bottom graph) Similarly, no significant increases in the frequency of cage crosses over a 30 min period were observed following ICI-118,551.

**FIGURE 6:**
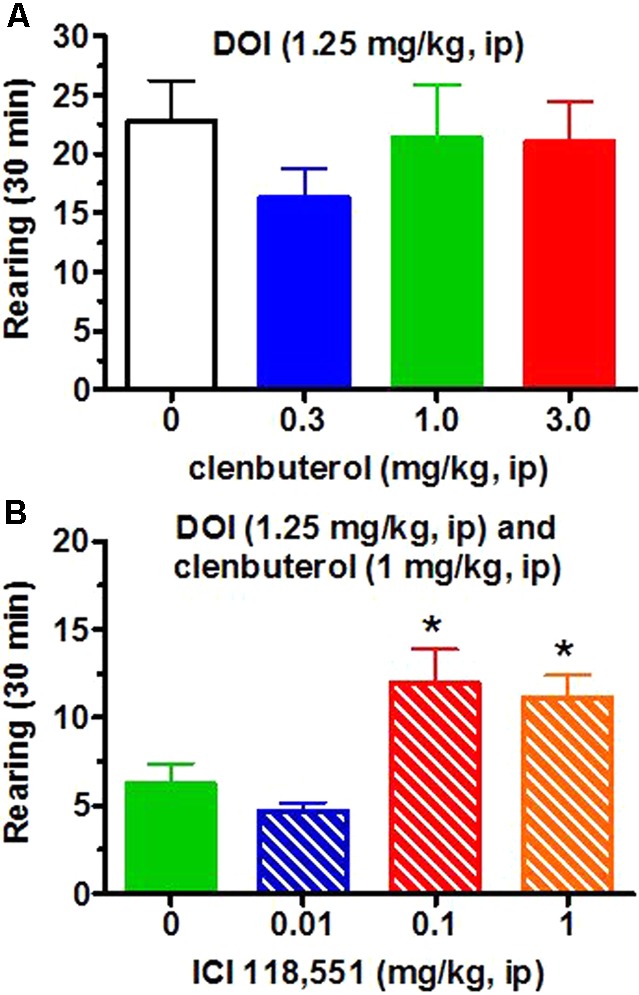
Clenbuterol (0.3–3.0 mg/kg, i.p.) did not alter the frequency of rearing. (**A**, top graph) Clenbuterol did not alter the frequency of rearing over a 30 min period in rats administered DOI. (**B**, bottom graph) ICI-118,551 (0.1–1.0 mg/kg) increased rearing episodes after clenbuterol (1.0 mg/kg)/DOI (1.25 mg/kg). ^∗^*p* < 0.05 for comparison to DOI/clenbuterol/ICI-118,551 vehicle.

The frequency of head twitches in vehicle-treated animals is less than 1.0 per 30 min observation periods (unpublished observations) while the frequency of DOI-induced head twitches in Sprague–Dawley rats generally ranges between 15 and 30 per 30 min observational periods. For the present experiments, DOI (1.25 mg/kg, i.p.; *n* = 12) induced approximately 25.9 ± 4.1 (SEM) head twitches/30 min. The β_2_-adrenergic receptor agonist clenbuterol (0.3–3 mg/kg, i.p.) suppressed DOI (1.25 mg/kg)-induced head twitches in a dose-dependent manner [*F*(3,33) = 3.58, *p* < 0.05; **Figure 4**]. The lowest effective clenbuterol dose (1.0 mg/kg) resulted in a similar effect (approximately 55% decrease) as the highest dose tested (3.0 mg/kg; Dunnett test, *p* < 0.05) compared to the frequency of head twitches induced by DOI (1.25 mg/kg) and the clenbuterol vehicle. These suppressant effects of clenbuterol on DOI-induced head twitches do not appear to be non-specific effects as clenbuterol did not alter the number of cage crosses [*F*(3,33) = 1.46, *p* > 0.2; **Figure 5**] or rearing [*F*(3,33) = 0.96; **Figure 6**] seen following the administration of DOI.

The selective β_2_-adrenergic receptor antagonist ICI-118,551 (0.01–1 mg/kg, i.p.) appeared to at least partially reverse the suppression by clenbuterol (1 mg/kg, i.p.) of DOI (1.25 mg/kg, i.p.)-induced head twitches in a dose-dependent manner [*F*(3,60) = 4.63, *p* < 0.01; **Figure 4**) in an entirely separate experiment (*n* = 16/each group) compared to the initial experiment demonstrating the suppressant action of clenbuterol (**Figure 4**, top graph). Thus, clenbuterol suppressed the number of DOI-induced head twitches by approximately 50% while ICI-118,551 (1 mg/kg) slightly more than doubled the number of DOI-induced head twitches observed following clenbuterol (1 mg/kg) in this second experiment with the antagonist. The highest ICI-118,551 dose tested significantly increased the number of DOI-induced head twitches after clenbuterol (1 mg/kg) compared to the suppressant effect of clenbuterol (1 mg/kg) alone on DOI-induced head twitches in this second experiment (Dunnett test, *p* < 0.05). ICI-118,551 had no effect on locomotor activity in rats administered DOI (**Figure 5**), though it did appear to increase rearing at the two higher doses (**Figure 6**).

## Discussion

The present results demonstrate that activation of β_2_-adrenergic receptors by clenbuterol suppressed head twitches induced by the phenethylamine hallucinogen DOI that are mediated by activation of 5-HT_2A_ receptors. The attenuation of the clenbuterol suppressant action on DOI-induced head shakes by the selective β_2_-adrenergic receptor antagonist ICI-118,551 is consistent with the known *in vivo* action of clenbuterol as a selective β_2_-adrenergic agonist. Given that intracerebral administration of 5-HT_2A_ agonists into the mPFC induces head twitches (Willins and Meltzer, 1997), these findings are consistent with the present observation that epinephrine selectively suppressed DOI-induced glutamate overflow when applied to rat prefrontal cortical slices. The pharmacological experiment with the β_2_-adrenergic agonist, ICI-118,551 and a previous report involving the cAMP transduction pathway (Lambe and Aghajanian, 2007) are consistent with an important modulatory action of β_2_-adrenergic receptor activation suppressing glutamate overflow induced by phenethylamine hallucinogens. However, further work will be required to confirm that these electrophysiological effects of epinephrine are mediated by β_2_-adrenergic receptors and not β_1_-adrenergic receptors.

### Novel Receptor and Potential Transduction Pathway Suppressing DOI Effects

**FIGURE 7:**
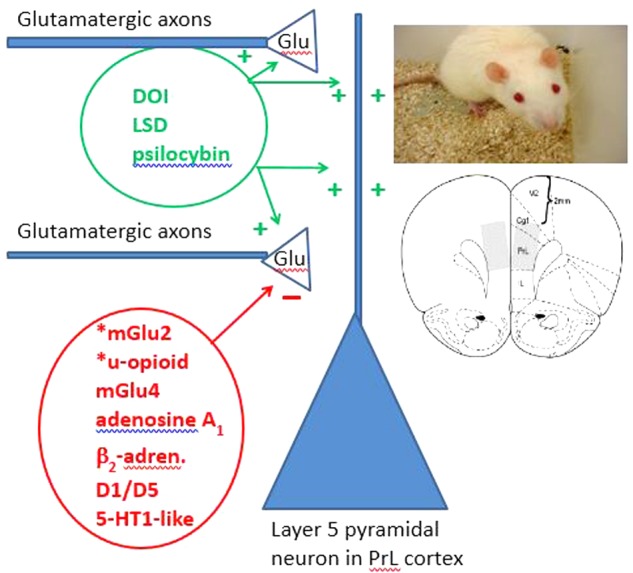
Model of glutamatergic input terminating in layers I and Va onto the apical dendrites of layer V pyramidal neurons of the prefrontal cortex (PFC) prelimbic region. Serotonergic hallucinogens (including DOI, LSD) increase glutamate overflow in layers I and Va of the PFC from glutamatergic terminals originating from the intralaminar and midline thalamic nuclei. 5-HT also increases this source of glutamate release. A number of G*_i_*/G*_o_*-coupled receptors are able to suppress this glutamate overflow induced by 5-HT_2A_ receptor activation including mGlu_2_, mGlu_4_, μ-opioid, adenosine A_1_, 5-HT_1-like_ receptors. Several canonical G*_s_*-coupled receptors (β_2_-adrenergic and dopamine D_1/5_ receptors) also suppress glutamate release from what may be the same afferents. The model shown assumes that all of these receptors are on the same terminals. Infusions of serotonergic hallucinogens into the mPFC (prelimbic region and anterior cingulate cortex) have been shown to induce the head twitch response in rodents (mPFC regions shown in right lower inset). The relationships shown for suppressing glutamate overflow onto layer V pyramidal cell apical inputs appear to also operate for suppression of the serotonergic hallucinogen-induced head twitch response in awake, behaving rodents (right top inset). ^∗^ Denotes those receptors shown to be decreased in layers I and Va of the rat PFC after midline thalamic lesions where an attenuation of 5-HT-induced spontaneous EPSCs is observed. In this model, there is no attempt to show the proportion of 5-HT_2A_ receptors localized to the post-synaptic side of glutamatergic terminals (a large majority in addition to other 5-HT_2A_ receptors localized to GABAergic interneurons and glial cells) compared to a putative minor population on the preterminal or terminal region of thalamocortical afferents.

The present results raise the question of whether a receptor with a novel transduction pathway suppresses DOI-induced head twitches. Previous work has demonstrated that a number of canonical G*_i_*/G*_o_*-coupled receptors suppress both the electrophysiological evidence for DOI-induced glutamate release and DOI-induced head twitches (Marek, 2017). This list of G-coupled receptors includes mGlu_2_, mGlu_4_, adenosine A_1_, and μ-opioid receptors, all which are known to have prominent or near-exclusive presynaptic actions in the CNS. In contrast, β_2_-adrenergic receptors are commonly coupled with G*_s_*-proteins where they increase production of cAMP as the initial step of their transduction pathway. While current data does not rule out a role for β_1_-adrenergic receptors in suppressing DOI-induced head twitches, β-adrenergic receptor activation has been associated with an increase in glutamate release from cerebrocortical synaptosomes via an increase on cAMP and downstream effects on transmitter release machinery (Ferrero et al., 2013). Activation of dopamine D_1/5_ receptors or application of forskolin or 8-Br-cAMP also suppressed apparent glutamate overflow induced by DOI in rat PFC (Lambe and Aghajanian, 2007). These observations, coupled with the current observations for epinephrine and a β_2_-adrenergic receptor antagonist are all consistent with the hypothesis that activation of β_2_-adrenergic receptors with the accompanying increases in cAMP suppress both the electrophysiological (late EPSPs) and behavioral (head twitches) effects of serotonergic hallucinogens (**Figure 7**).

The known regional and cellular distribution of these receptors suppressing DOI-induced glutamate overflow or DOI-induced head twitches is consistent with the importance of thalamocortical pathways in mediating these effects. The frequency of 5-HT-induced EPSCs is decreased by extensive thalamic lesions that damage the medial and intralaminar thalamic nuclei (Lambe and Aghajanian, 2001; Marek et al., 2001). These thalamic lesions were also found to decrease the amount of mGlu_2/3_ receptor or μ-opioid receptor binding in layers I and Va of the PFC (Marek et al., 2001), the cortical laminae with the richest innervation arising from these so-called non-specific thalamic nuclei (Berendse and Groenewegen, 1991). In the rat, β_2_-adrenergic receptors appear to be enriched in layers I and Va of the PFC/neocortex, although this mid-cortical enrichment is sometimes suggested to be layer IV (Rainbow et al., 1984; Booze et al., 1989). Accordingly, mRNA *in situ* hybridization studies have found a moderate β_2_-adrenergic receptor mRNA labeling in the midline and intralaminar thalamic nuclei in contrast to a relative lack of prefrontal cortical/neocortical labeling (Nicholas et al., 1993). The highest β_2_-adrenergic receptor mRNA labeling in the thalamus is for the midline and intralaminar thalamic nuclei which project to layer I and Va throughout the PFC/neocortex. A lesion study has verified that thalamic lesions decrease the amount of β_2_-adrenergic receptor binding in the PFC/neocortex (Vogt et al., 1992). Thus, β_2_-adrenergic receptors appear to be similarly located within thalamocortical afferents arising from the medial and intralaminar thalamic nuclei similar to mGlu_2_ and μ-opioid receptors.

### Therapeutic Implications of Clenbuterol Suppression of DOI-Induced HTR

As discussed above, a range of agonists or positive allosteric modulators that activate G*_i_*/G*_o_*-coupled receptors and β_2_-adrenergic receptor agonists appear to both suppress the electrophysiological effects of 5-HT or serotonergic hallucinogens in additional to suppressing DOI-induced head twitches (Marek, 2017). While DOI-induced head shakes or the HTR has been used as one component of a battery of paradigms screening for potential novel antipsychotic drugs, many antidepressant drugs also suppress DOI-induced head twitches. This primarily includes most tricyclic antidepressants and heterocyclic antidepressants (mirtazapine, mianserin, trazodone, and nefazodone) that all potently block 5-HT_2A_ receptors). Modulation of 5-HT_2A_ receptor responses in the PFC may be a key feature in detecting novel antidepressant drugs with serotonergic hallucinogen-induced head twitches. Interestingly, mGlu_2_ receptor PAMs, adenosine A_1_ receptor agonists, and β_2_-adrenoceptor agonists all induce antidepressant-like activity when administered to rodents trained on an operant screen for antidepressant drugs, the DRL 72-s schedule of reinforcement (O’Donnell, 1987; Nikiforuk et al., 2010; Fell et al., 2011; Marek, 2012). While an mGlu_2_ receptor PAM-tested in MDD did not result in clear antidepressant effects in a phase 2 trial, there are several small positive clinical trials with the β_2_-adrenergic receptor agonist salbuterol in depressed patients (Lecrubier et al., 1980; Simon et al., 1984). These results raise the possibility that the distribution of receptors suppressing DOI-induced head shakes by virtue of localization within critical microcircuitry (e.g., prefrontal cortical layer V pyramidal cells) might sculpture the phenotype of associated behavioral effects toward those with antidepressant-like and/or antipsychotic-like profiles.

The pro-cognitive profile of β_2_-adrenergic receptor agonists in primates and rodents raises the possibility that addition of β_2_-adrenergic receptor agonists to ongoing treatment with SSRIs may enhance antidepressant efficacy but also improve cognitive dysfunction associated with MDD. Specifically, either direct prefrontal infusions or systemic administration of clenbuterol to rats or primates improved working memory performance (Ramos et al., 2008). Speaking to differential effects of β-adrenergic receptor subtypes on cognition, either direct prefrontal infusions or systemic administration of the β_1_-adrenergic receptor antagonist betaxolol also improved the working memory of rodents or non-human primates (Ramos et al., 2005). These opposing actions of β-adrenergic receptor subtypes on cognition are consistent with the differential distribution of β-adrenergic receptor subtypes. The β_2_-adrenergic receptor protein and mRNA distribution was described above while β_1_-adrenergic receptor protein and mRNA are moderately localized throughout the PFC/neocortex and is also present in GABAergic cells of the reticular nucleus of the thalamus. This differential distribution of β-adrenergic receptor subtype protein and mRNA in thalamocortical circuits may serve as a foundation for the opposing actions of β-adrenergic receptor subtypes on executive functions reflecting prefrontal cortical function (Ramos and Arnsten, 2007). These relationships may be especially important when considering unmet medical needs of cognitive impairment in the treatment of major depressive disorder (MDD), including residual cognitive dysfunction following remission of other MDD symptoms.

### Limitations

A primary limitation in the interpretation of the results described here is that further electrophysiological studies directed at understanding the role of β_2_- and β_1_-adrenergic receptors in thalamocortical transmission are required that could also help determine the actual source of stimulated afferents to layer V pyramidal neurons. Furthermore, exploration with a wider range of β_1_-adrenergic and β_2_-adrenergic receptor antagonists would be one strategy to confirm whether there is an exclusive role for β_2_-adrenergic receptors in mediating the electrophysiological effects of epinephrine described here. Second, additional studies are required to determine whether other effects of clenbuterol or ICI-118,551 might also play a role in the behavioral effects. While ICI-118,551 at a 1 mg/kg dose did not reverse DOI-induced head twitches following clenbuterol to the same absolute magnitude of head twitches following DOI in the initial experiment with clenbuterol, the most likely explanation for this effect may be the two- to three-fold variability in the number of DOI-induced head twitches observed with independent cohorts of Sprague–Dawley rats. Accordingly, clenbuterol (1 mg/kg) suppressed DOI-induced head twitches by approximately 50% in one experiment while ICI-118,551 nearly doubled the frequency of head twitches observed after the administration of both DOI and clenbuterol (1 mg/kg) in another experiment. Third, an important step forward for the behavioral studies would be to extend these results toward an exploration of the 2nd messenger pathways involved. Fourth, these results bringing forth an additional class of receptor and GPCR-protein interactions with cortical 5-HT_2A_ receptors would appear to challenge the view that these functional interactions between a number of G*_q_*-coupled receptors inducing glutamate release and a number of receptors suppressing glutamate release can be explained simply by heteromeric receptor complexes (Gonzalez-Maeso et al., 2008; Moreno et al., 2011, 2012). Rather, the growing number of receptor interactions would suggest that simple functional interactions may play the primary role in these effects since evidence has only been advanced for interactions of the 5-HT_2A_ receptor with mGlu_2_ or mGlu_4_ receptors (Delille et al., 2013; Marek, 2017). Alternatively, heteromeric complexes between 5-HT_2A_ and mGlu_2_ receptors and simple functional interactions could both be relevant for these electrophysiological and behavioral relationships.

## Conclusion

Activation of β_2_-adrenergic receptors appears to suppress DOI-induced head twitches. This behavior appears to involve the functional output of layer V pyramidal cells in the prelimbic region of the mPFC and the anterior cingulate. This novel finding for the β_2_-adrenergic receptor adds a G_s_-type of protein-coupled receptor (β_2_-adrenergic) alongside a number of G*_i_*/G*_o_*-coupled receptors (mGlu_2_, mGlu_4_, adenosine A_1_, u-opioid, 5-HT_1A_) that similarly act to suppress both the electrophysiological and a psychotomimetic-like behavioral effect of serotonergic hallucinogens in the mPFC. These relationships are consistent with clinical data supporting antipsychotic activity for mGlu_2_ and μ-opioid receptor agonists (and 5-HT_2A_ receptor antagonists) and other clinical evidence suggesting antidepressant efficacy for 5-HT_1A_, β_2_-adrenergic, μ-opioid receptor agonists (Carlson and Simpson, 1963; Lecrubier et al., 1980; Simon et al., 1984; Bodkin et al., 1995; Marek and Aghajanian, 1998b; Patil et al., 2007; Kishi et al., 2014; Kinon et al., 2015).

## Author Contributions

GM and BR designed the research, performed the experiments, and analyzed and discussed the data. GM wrote the manuscript.

## Conflict of Interest Statement

A portion of the behavioral studies (involving the β_2_-adrenergic receptor antagonist ICI-118,551) were conducted while GM was initially employed by Pfizer. The other author declares that the research was conducted in the absence of any commercial or financial relationships that could be construed as a potential conflict of interest.
